# Primary Reconstruction of Extended Multifragmented Skull Fracture: Case Report and Technical Note

**DOI:** 10.3390/reports8030102

**Published:** 2025-06-26

**Authors:** Iván N. Camal Ruggieri, Guenther C. Feigl, Gavin W. Britz, Dzmitry Kuzmin, Daniel Staribacher

**Affiliations:** 1 Department of Neurosurgery, General Hospital Bamberg, 96049 Bamberg, Germanydaniel@drstaribacher.at (D.S.); 2 Department of Neurosurgery, University Hospital Tuebingen, 72016 Tuebingen, Germany; 3 Department of Neurological Surgery, Houston Methodist Hospital, Houston, TX 77030, USA

**Keywords:** skull, reconstruction, traumatic brain injury

## Abstract

**Background and Clinical Significance:** Traumatic brain injury (TBI) represents a major public health concern due to its profound neurological, psychological, and socioeconomic consequences. Effective management is essential to optimize patient outcomes and reduce healthcare burden. In cases involving extensive bone loss or complex fractures, particularly when decompressive craniectomy (DC) is considered, secondary cranial reconstruction is typically required. However, DC is associated with prolonged hospitalization, multiple surgical interventions, an increased risk of complications, and higher costs. **Case Presentation:** We present the case of a 59-year-old male involved in a high-energy bicycle accident, sustaining severe craniofacial trauma with multiple midface fractures, a multifragmented left cranial fracture, and a left-sided epidural hematoma with brain compression. Hematoma evacuation and immediate primary reconstruction of the fractured skull using autologous bone were successfully performed, avoiding the need for DC. The patient recovered under intensive care and was transferred to a neurorehabilitation center. **Conclusions:** Primary reconstruction of large skull fractures using autologous bone should remain the goal, whenever possible, in order to avoid additional costs, risks, and complications.

## 1. Introduction and Clinical Significance

Traumatic brain injury (TBI) represents a significant public health challenge, affecting not only patients and their families through a range of psychiatric, psychological, emotional, cognitive consequences, and neurological deficits but also society at large, with substantial economic implications. An estimated 50 to 60 million TBIs occur annually worldwide, with mortality rates for severe TBI ranging from 30 to 40% [1,2,3].

Therefore, optimal management of TBI is of paramount importance—not only to the patient and family, but also to society. Currently, TBI is managed by a combination of conservative medical and surgical strategies, ideally followed by rehabilitation to promote the patient’s recovery and social reintegration. However, current international surgical guidelines are limited by a lack of robust evidence. It is unclear which populations benefit from different surgical procedures or whether the timing of surgery is optimal. Furthermore, there are numerous external factors that influence the decision to operate, such as outcome expectations, family preferences, and the experience of the surgical team, among others.

Decompressive craniectomy (DC) is a last-resort surgical procedure that may be employed in cases of severe TBI when other medical interventions prove inadequate. The procedure is designed to reduce intracranial hypertension, a condition that can result in significant morbidity and mortality if left untreated. Nevertheless, it is associated with a multitude of complications, and the necessity of the procedure must be carefully considered in light of the potential risks and benefits [4,5,6,7].

The DECRA (Decompressive Craniectomy in Diffuse Traumatic Brain Injury) study, a randomized controlled trial (RCT), demonstrated inferior outcomes in patients with elevated intracranial pressure (ICP) and very early DC. The RESCUEicp (Randomized Evaluation of Surgery with Craniectomy for Uncontrollable Elevation of Intracranial Pressure trial) study, another RCT, indicated that DC enhanced survival, albeit with significant patient dependence [8,9].

In view of the currently available evidence, it is challenging to determine the optimal surgical treatment approach for severe TBI. Similarly, a decision regarding surgical management must be made in cases of complex multifragmented skull fractures. One may opt to perform a craniectomy of considerable magnitude, which does not necessarily entail a hemicraniectomy and involves the removal of fracture fragments and subsequent reconstruction in a secondary operation. An alternative approach would be to attempt a primary reconstruction of the fracture. In cases where there is evidence of potential secondary injury due to diffuse edema and elevated ICP, a decompressive hemicraniectomy is clearly indicated. In the absence of evident indications, the responsibility for determining the optimal course of action rests on the surgical team.

In the aftermath of either a craniectomy or a decompressive hemicraniectomy, the subsequent cranial reconstruction must be conducted with a biocompatible and biologically inert material. Furthermore, the material must possess osteoinductive and osteoconductive properties and should also be resistant to inflammatory reactions and external mechanical forces [10]. Moreover, the material must be readily manipulable and have minimal societal and economic cost implications. It is regrettable that no material currently exists that meets all of the aforementioned characteristics. Nevertheless, there is currently a wide range of materials (autologous bone, hydroxyapatite, titanium, polymethylmethaacrylate, and polyetheretherketone) with varying advantages and disadvantages for craniectomy reconstruction. Thus far, no material has been shown to have a definitive advantage over the others [11,12,13,14,15,16].

Nevertheless, given its biological compatibility, low cost, and minimal risk of rejection, autologous cranial bone remains the optimal graft for reconstruction in small and medium-sized defects. The selection of the appropriate material should be based on the individual patient, taking into account a number of factors, including the size of the decompression, the age of the patient, the existence of comorbidities, the probability of recovery, and the expectations of a favorable long-term outcome. In addition, a cost analysis should be conducted.

Given the clinical and economic significance of these procedures, as well as the dearth of evidence and recommendations pertaining to complex multifragmentary fractures in the absence of decompressive hemicraniectomy, we undertook this technical note to contribute to the evidence base for the operative management of severe TBI.

## 2. Case Presentation

**Figure 1:**
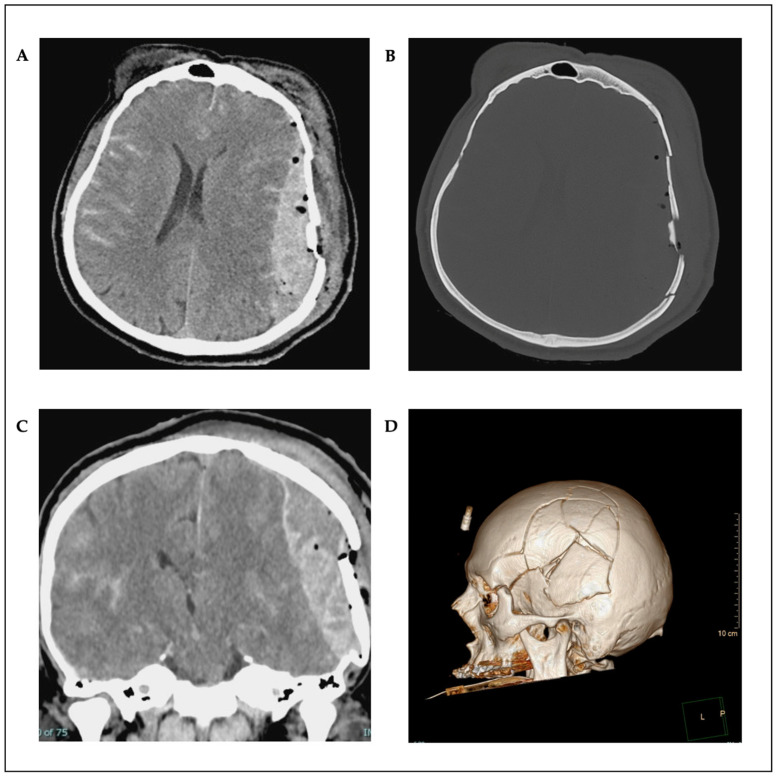
Preoperative axial (**A**,**B**) and coronal (**C**) CT scans; 3D reconstruction (**D**).

A 59-year-old male without pre-existing conditions was involved in a vehicular accident. He was riding an e-bike and collided with an oncoming vehicle. He sustained a direct impact to the forehead, resulting in contact with the dashboard. The left side of the skull was exposed and exhibited evidence of contusion. The head, forehead, and face exhibited a multitude of lacerations. Both eyes exhibited edema, and cerebrospinal fluid (CSF) was observed to be leaking from the nose with the presence of blood and active bleeding from the ears. The left ear showed a near-total rupture with a pendulous distal portion. No additional injuries were identified. The patient presented with confusion and agitation, requiring immediate intubation to secure the airway due to the severity of the head injury. The patient was transported to our facility via helicopter. The computed tomography (CT) scan revealed a brain injury with a fractured skull on the left side and an epidural hematoma on the left side with a midline shift and compression of the brain. Furthermore, the scan demonstrated the presence of a left parietal hemorrhagic contusion, bilateral subarachnoid hemorrhage (SAH), and multiple midface fractures (Figure 1A–D). The patient was then transferred to the operating room, where surgical intervention was performed. The epidural hematoma was drained, and the skull fracture was reconstructed with plates and screws.

**Figure 2:**
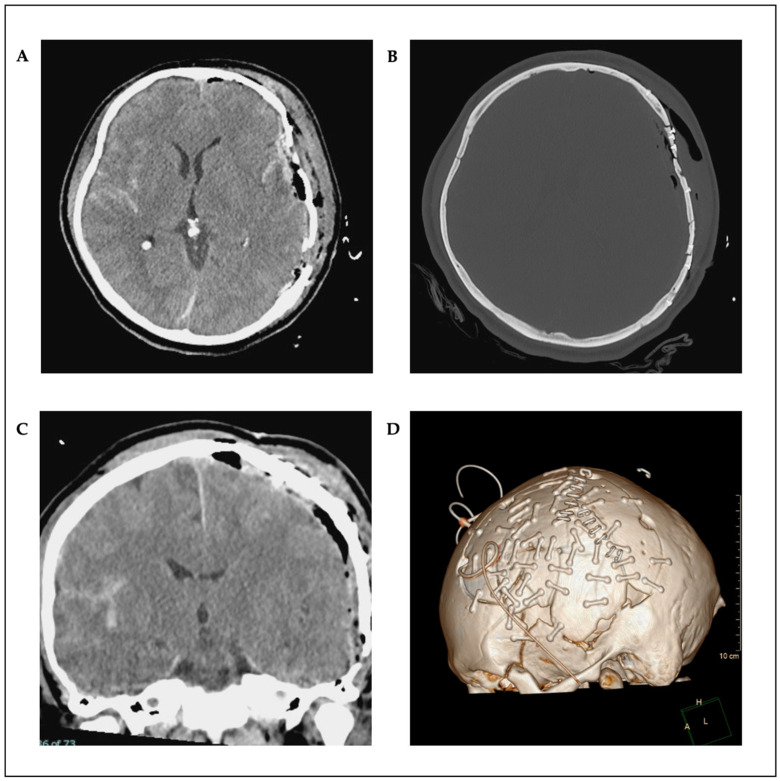
Postoperative axial (**A**,**B**) and coronal (**C**) CT scans; as well as 3D reconstruction (**D**).

Postoperative CT scan showed a complete evacuation of the epidural hematoma with a regressive midline shift (Figure 2A–D). Osmodiuretic mannitol and thiopental were temporarily administered in response to pathologically elevated ICP values. This therapy was then discontinued, and a magnetic resonance imaging (MRI) scan showed no correlation with edema or axonal diffuse injury. The ENT (Ear–Nose–Throat) surgeon performed a Le Fort III fracture repair using a supraorbital approach on both sides. Sedative medications were progressively tapered on a daily basis. Prolonged sedation led to pronounced critical illness myopathy, although no paralysis was observed. The patient was then transferred to a neuro-rehabilitation center. Following neurorehabilitation, the patient showed neurological improvement with no motor deficits and no significant cognitive deficits, except for mild short-term memory impairment at 12-month follow-up. Moreover, there were no signs of delayed wound healing, wound defects, or infection. He was able to reintegrate into his job and daily life.

### Surgical Technique

**Figure 3:**
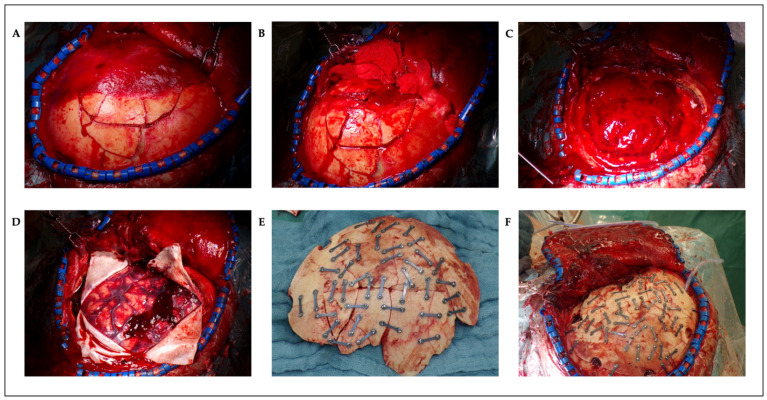
Intraoperative photos: multifragmented skull fracture of the left frontotemporoparietal region with attached temporal muscle (**A**), after detachment of the temporal muscle (**B**), epidural hematoma (**C**), minimal subdural bleeding (**D**), autologous reconstructed fracture fragments on table (**E**), and autologous reconstructed fracture in situ (**F**).

The patient was positioned supine. A Mayfield head-clamp was placed, and the head was slightly rotated to the right. The torn ear was temporarily refixed with single-button sutures. A trauma flap skin incision was made on the left side. Raney clips were placed, and the fracture and bone fragments were carefully exposed (Figure 3A). Some of the bone fragments were removed from the surgical wound using forceps. Subsequently, the temporal muscle was carefully elevated. Fractures extending up to the skull base were exposed (Figure 3B). A craniotomy was created around the fracture, first by making burr holes and then connecting them with the craniotome. The fracture was lifted in several bone fragments. An organized hematoma was identified underneath (Figure 3C) and was carefully removed with a dissector under continuous irrigation. Bleeding, especially from the area of the middle meningeal artery, was coagulated. Small tears in the area of the dura at the base were visualized. After complete evacuation of the hematoma, the dura was fully exposed. The dura was then opened in a crosswise fashion. A minimal subdural blood deposit was identified (Figure 3D) and was softly rinsed off. The brain appeared partially contused but showed slight pulsations. Repeated rinsing was performed. As no further space-occupying bleeding was present, the dura was closed with watertight sutures and sealed with TachoSil^®^ (Corza Medical GmbH, Düsseldorf, Germany). The bone fragments were then reconstructed using small titanium plates and screws (MatrixNEURO™ Sterile Kit, DePuy Synthes GmbH, Oberdorf, Switzerland) and were repositioned *en bloc* to their original position (Figure 3E,F). Dura tenting sutures were placed and threaded through the fixed bone flap. A Redon drain was inserted epidurally. The wound was then closed in layers. The temporal muscle was sutured to the bone fragments using small bone plates. A subcutaneous suture was placed, followed by a penetrating skin suture. An ICP catheter was placed contralaterally for postoperative monitoring.

## 3. Discussion

The management of multifragmented skull fractures in TBI remains complex, especially when deciding between DC and primary reconstruction. In this case, primary reconstruction using autologous bone fragments secured with titanium plates was chosen due to the absence of severe brain edema and manageable ICP. This approach offered several advantages, including avoiding the risks associated with DC, such as secondary surgeries, infection, and prolonged recovery. Autologous bone remains the preferred material for reconstruction due to its biological compatibility and reduced rejection risk. Although DC is necessary in cases of uncontrolled ICP, primary reconstruction offers a safe alternative in selected patients, particularly when secondary injury risk is low. Despite the benefits of primary reconstruction, potential complications such as bone resorption, pressure ulcers, skin necrosis, wound dehiscence, and infection must always be considered, particularly in cases requiring multiple fixation devices.

Nonetheless, alternative materials such as a full titanium mesh should also be considered, as they carry similar complication rates—such as pressure ulcers, skin necrosis, wound dehiscence, and infection—but may offer improved outcomes regarding bone resorption, albeit at a higher financial cost [17]. This case illustrates that early reconstruction can lead to better cosmetic and functional outcomes, shorter hospital stays, and lower healthcare costs. The favorable outcome, despite postoperative critical illness myopathy, supports the idea that individualized surgical planning—based on intraoperative findings—can optimize patient recovery in severe TBI cases.

## 4. Conclusions

Primary skull reconstruction using autologous bone is a viable alternative to DC in select TBI cases with stable ICP. This approach reduces the need for secondary surgeries, complications, and healthcare costs, while improving patient outcomes. Although DC remains essential for cases with severe cerebral edema, primary reconstruction should be considered when conditions allow, as it can lead to better recovery and resource optimization.

## Data Availability

The original contributions presented in this study are included in the article. Further inquiries can be directed at the corresponding authors.

## Abbreviations

The following abbreviations are used in this manuscript:

TBI	Traumatic brain injuries
DC	Decompresive craniectomy
DECRA	Decompresive craniectomy in diffuse traumatic brain injury
RCT	Randomized controlled trial
ICP	Intracranial pressure
RESCUEicp	Randomized Evaluation of Surgery with Craniectomy for Uncontrollable Elevation of Intracranial Pressure
CSF	Cerebrospinal fluid
CT	Computed tomography
SAH	Subarachnoid hemorrhage
MRI	Magnetic resonance imaging
ENT	Ear-Nose-Throat
